# Detecting the effects of selection at the population level in six bovine immune genes

**DOI:** 10.1186/1471-2156-9-62

**Published:** 2008-10-06

**Authors:** Abigail R Freeman, David J Lynn, Caitriona Murray, Daniel G Bradley

**Affiliations:** 1 Smurfit Institute of Genetics, Trinity College, Dublin 2, Ireland

## Abstract

**Background:**

The capacity of a species or population to respond to and survive novel infectious disease challenge is one of the most significant selective forces shaping genetic diversity and the period following animal domestication was likely one of the most important in terms of newly emerging diseases. Inter-specific genome-wide comparison has suggested that genes, including cluster of differentiation 2 (CD2), ADP-ribosyltransferase 4 (ART4), tyrosine kinase binding protein (TYROBP) and interleukins IL2, IL5, IL13, may have undergone positive selection during the evolution of the bovine lineage. Past adaptive change implies that more recent variation may have also been subject to selective forces.

**Results:**

In this paper, we re-sequence each of these genes in cattle cohorts from Europe, Africa and Asia to investigate patterns of polymorphism at the population level. Patterns of diversity are higher within *Bos indicus *suggesting different demographic history to that of *Bos taurus*. Significant coding polymorphism was observed within each of the cell-surface receptors. In particular, CD2 shows two divergent haplotypes defined by a series of six derived nonsynonymous substitutions that are significantly clustered on the extracellular surface of the protein and give significant values for Fay and Wu's *H*, strongly suggesting a recent adaptive history. In contrast, the signaling molecules (especially IL13) display outlying allele frequency spectra which are consistent with the effects of selection, but display negligible coding polymorphism.

**Conclusion:**

We present evidence suggestive of recent adaptive history in bovine immune genes; implying some correspondence between intra- and inter-specific signals of selection. Interestingly, three signaling molecules have negligible nonsynonymous variation but show outlying test statistics in contrast to three receptors, where it is protein sequence diversity that suggests selective history.

## Background

Domestication has involved severe and novel selective pressures on cattle and other domesticates, undoubtedly leaving behind population genetic signatures of adaptation [1]. In particular, the epidemiology of infectious diseases would have changed during domestication due to sharp increases in population densities and the new proximity of previously separated species. The effect of the domestication process in terms of emerging diseases is often discussed with respect to human populations [2], however, similar processes are as likely to have shaped the genomes of domesticated animals. By screening the bovine genome for selective signatures associated with immunity or disease susceptibility, we may be able to identify those genes that have been of critical importance to the development of disease resistance [3].

We have previously reported a comparative genomics study identifying several bovine genes, including cluster of differentiation 2 (CD2) and ADP-ribosyltransferase 4 (ART4), as having significant evidence of adaptive evolution on the evolutionary lineage leading to modern cattle from the bovine-pig common ancestor [4]. Briefly, we investigated evidence of variable selective pressure on the bovine lineage in a dataset of approximately 3,000 orthologs from human, mouse, cow and pig. A gene was inferred to be subject to adaptive evolution on the bovine lineage when a model of variable selective pressure specifically on that lineage was significantly favoured over the alternative model and the ratio of nonsynonymous substitutions per nonsynonymous site to synonymous substitutions per synonymous site, ω, was significantly greater than 1. CD2 (ω = 3.858) and ART4 (ω = 1.356) were ranked first and third in a list of significant scores ranked by this estimated ratio.

Emerging data from the bovine genome project has allowed us to similarly investigate a larger number of bovine genes. Interleukins 2, 5, 13 (IL2, IL5 and IL13), and tyrosine kinase binding protein (TYROBP) each gave preliminary evidence suggestive of adaptive evolution on the bovine lineage (Lynn et al, unpublished).

Several studies of human genes, which have signatures of adaptive evolution between species, found that many of these genes have also been subject to more recent population selection [5-7]. To assess the level of within-species polymorphism and to identify potential population-specific selective signatures in these six bovine immune genes we have re-sequenced exonic, intron and intergenic regions of each in population samples from Africa, Asia and Europe, plus an outgroup species, bison. Thirty-nine individual cattle, representing 16 different breeds were included in these samples. The three continental groups, which probably reflect the products of separate domestication events, certainly have had different post-domestic histories and have endured markedly different infectious challenges [8,9].

## Results

### Patterns of sequence diversity

The three samples chosen for re-sequencing were multi-breed collections of European and West African *Bos taurus *plus *B. indicus *sampled across four breeds of South Asian origin. Each of these three continental populations is thought to have separate histories since domestication and here the legacy of migration was minimized by careful selection from populations known from prior data to have experienced minimal introgression [10]. Summary statistics and tests of neutrality are given for each gene within each population sample in Table 1. Each locus showed polymorphism, within and between the three continental samples (alignments are given in Additional file 1). Interestingly neither IL2 nor IL5 showed any nonsynonymous sequence changes and IL13 displayed only a single nonsynonymous change present in one African and four *B. indicus *chromosomes. There is a marked tendency for the *B. indicus *sample to give the highest sequence diversity (five from six loci) and also the highest haplotype diversity (five from six loci).

**Table 1:** Neutrality statistics for six genes re-sequenced in European and African *B.taurus *and *B.indicus *cattle

**Gene name**	**N**	**S**	**No.Haplotypes**	**Hd**	**θ (persite)**	**π (persite)**	**Tajima's *D***	**Fu & Li's *D***	**Fu & Li's *F***	**Fay & Wu's *H***	**Fu's *FS***
**ART4**											
*Africa*	20	6	6	0.747	0.0008	0.0010	0.721	-0.239	0.048	0.779	-0.168
*Europe*	36	6	6	0.783	0.0007	0.0006	-0.146	1.234	0.953	0.397	-0.481
*Bos indicus*	22	33	16	0.961	0.0042	0.0046	0.332	1.747**	1.549*	-2.338	-3.026
**CD2**											
*Africa*	20	13	5	0.616	0.0010	0.0010	0.051	1.193	1.008	-1.2	2.696
*Europe*	36	30	10	0.846	0.0019	0.0016	-0.576	0.611	0.221	-9.511*	1.765
*Bos indicus*	22	29	10	0.87	0.0021	0.0023	0.236	1.2	1.068	-7.775	1.297
**TYROBP**											
*Africa*	20	8	6	0.779	0.0006	0.0006	-0.137	0.799	0.62	-3.779*	-0.05
*Europe*	36	20	12	0.657	0.0012	0.0006	-1.607*	-1.193	-1.614	-7.356**	-3.455*
*Bos indicus*	22	12	11	0.913	0.0008	0.0010	0.745	0.629	0.799	1.887	-2.192
**IL2**											
*Africa*	20	1	2	0.268	0.0001	0.0001	-0.086	0.627	0.512	-1.253	0.381
*Europe*	36	12	9	0.614	0.0008	0.0004	-1.754*	-1.144	-1.613	-5.680*	-3.667*
*Bos indicus*	22	7	7	0.593	0.0006	0.0004	-0.769	-1.479	-1.507	0.156	-2.008
**IL13**											
*Africa*	20	15	9	0.653	0.0013	0.0005	-2.156***	-3.521***	-3.731***	1.621*	-3.877**
*Europe*	36	14	13	0.814	0.0011	0.0008	-0.998	-2.034*	-2.02	1.756	-4.530*
*Bos indicus*	22	14	12	0.935	0.0012	0.0014	0.741	1.232	1.296	2.13	-2.505
**IL5**											
*Africa*	20	5	6	0.737	0.0008	0.0005	-1.298	1.133	0.495	12.547***	0.188
*Europe*	36	3	9	0.621	0.0009	0.0007	-0.729	0.823	0.350	-12.400**	-0.288
*Bos indicus*	22	4	10	0.753	0.0009	0.0008	-0.563	0.459	0.159	-3.151	-1.826

*/**/***indicate values that fall in a 0.05/0.01/0.001 negative tail under a standard coalescent simulation. Tajima's *D *and Fay and Wu's *H *values that fall in a 0.05 negative tail under a domestication simulation are indicated in bold type.

### Neutrality tests at each locus

#### Interleukin 13

Interleukin 13 (IL13) is a cytokine which plays a critical role in allergic inflammation and in susceptibility to parasites, such as, helminths, schistosomes and nematodes [11-14]. The pattern of diversity at the IL13 locus is consistent with non-neutral evolution in the African population sample, with significant results in four tests of neutrality (Table 1). Significantly negative values of Tajima's *D*, Fu and Li's, *D *and *F *statistics, and Fu's *Fs *were obtained for this population, suggesting positive selection; nucleotide diversity was also somewhat reduced relative to the other samples [15-17]. These significance values are calculated on coalescent simulations assuming a constant effective population size. In order to consider the demography of domestication, which probably included a significant post-domestic population expansion, we also calculated Tajima's *D *and Fay and Wu's *H *conditioned on a significant population expansion. The IL13 African *D *value remained below the 5^th ^percentile. Aside from the relatively robust signature of positive selection in the African population, Fu and Li's *D *and Fu's *Fs *also showed somewhat less significant departures from neutrality in European samples (Table 1).

IL13 displays almost no coding sequence polymorphism with only a single (nonsynonymous) SNP in some *B. indicus *samples and one African heterozygote (possibly due to *B. indicus *introgression). The nonsynonymous SNP (A7T) is not predicted to have an affect on protein structure by the SIFT or PolyPhen algorithms [18,19]. Apart from one synonymous substitution compared to plains bison, there are no differences between the bovine IL13 coding sequence and this outgroup species, although there are several intergenic and intronic SNPs.

#### Interleukin 5

This gene is part of a cytokine cluster on Bta7 along with IL13 and IL4, all with known involvement in the Th2-type response. Co-expression at this cluster is controlled by elements over a 120 kb range in humans [20]. The gene product is a main regulator of eosinophil maturation and activation and has been implicated as influencing *Shistosoma *infection in humans [21]. The gene showed moderate nucleotide and haplotypic diversity but no nonsynonymous polymorphisms within the sampled populations. Fay and Wu's *H *tests were significant in both the African (P = 0.001) and European (P = 0.002) samples and remained outside the 95% interval in comparison to values generated under a simulated domestication bottleneck and expansion.

#### Interleukin 2

IL2 is a central regulator of the immune system involved in processes including production of cytotoxic T-cells, proliferation of immunoglobulins by B-lymphocytes and induction and maintenance of natural killer (NK) cells [22]. Three neutrality tests, based on the allele frequency spectrum (Tajima's *D*, Fay and Wu's *H *and Fu's *Fs*), show significant deviation from neutrality at the IL2 locus in European populations (Table 1) [15-17,23]. On comparison to the coalescent model, modeled on a population expansion akin to domestication, Fay and Wu's *H *retained an outlying value (P < 0.025) but Tajima's *D *did not retain significance.

The African population contains only a single SNP over the 3461 bp re-sequenced at this locus; the lowest level of sequence diversity (0.0001) within any locus-sample combination (Table 1). No nonsynonymous polymorphism was observed at this locus.

#### Tyrosine Kinase Binding Protein

Tyrosine kinase binding protein (TYROBP; also known as DAP12) is involved in the activation of Natural Killer (NK) cell anti-viral responses, via 'non-inhibitory' members of killer-cell inhibitory receptor (KIR) family [24-27]. NK cell KIRs have been shown to be rapidly evolving in a wide range of species, including cattle, and have undergone lineage-specific expansions or contractions of the gene family [28-30]. It is tempting to speculate that adaptive evolution of bovine TYROBP, may be driven by co-evolution with rapidly evolving members of the KIRs, with which it interacts.

The results of the standard neutrality tests at this locus are not as clear as for the cytokines discussed previously (Table 1). Fu's *Fs *test [17] and Tajima's *D *are significantly negative in the European population, and Fay and Wu's *H *test [23] suggests an excess of derived SNPs in the African and European samples. Significance at the *H *test proved robust to a population expansion model but the *D *value fell within the 95% confidence interval.

TYROBP has two nonsynonymous SNPs within cattle, one only within the African sample (S12C) and one derived variant which is almost fixed within both the African and European samples (Q24L). Both of these occur in the signal peptide and are predicted by SIFT to have an impact on protein structure and/or function [18]. An additional nonsynonymous substitution (P106A), predicted by PolyPhen as 'possibly damaging', occurs between cattle and plains bison, and is located in the cytoplasmic domain of TYROBP.

#### ADP-ribosyltransferase 4

Human ADP-ribosyltransferase 4 (ART4) is an erythrocyte glycoprotein that has been identified as the Dombrock blood group [31]. The two major antigens of this blood group (Do^a ^and Do^b^) have significantly different frequencies in human populations and are associated with severe transfusion reactions [32]. Three other high incidence antigens (Gy^a^, Hy and Jo^a^) are also carried on the Dombrock blood group molecule.

Bovine ART4 was found to have five nonsynonymous and six synonymous SNPs within cattle populations and one nonsynonymous and synonymous substitution compared to plains bison. More specifically, four of the five nonsynonymous changes (G5R, R41M, I130V, S213A) and all of the within-cattle synonymous changes occur only in the *B. indicus *sample. Indeed, the *B. indicus *population exhibits much higher nucleotide diversity with 33 segregating SNPs compared to just 6 within each of the African and European populations (Table 1).

Cattle do not appear to be polymorphic at the orthologous position to the human mutation (N265D) which is responsible for the Do^a^/Do^b ^antigenicity. Bovine ART4 is, however, polymorphic at the position associated with the Jo^a ^antigen in humans (human T117I; bovine I130V) [32]. The antigenicity associated with this polymorphism in humans suggests that mutations at this position may result in structural alteration of the molecule. Two of the other nonsynonymous changes (G5R, G126W) are also predicted to alter protein structure by SIFT [18]. The I130V and G5R mutations are found only in *B. indicus *cattle while G126W is found only in African samples.

#### Cluster of Differentiation 2

Antigen recognition by T cells is one of the crucial steps in controlling adaptive immune responses. Cluster of Differentiation 2 (CD2) is a T cell and natural killer cell surface protein that optimizes T cell activation through interaction with its counter receptor; CD58 in non-rodent mammals [33-35] and CD48 in rodents [36]. CD2 and other similar cell surface proteins have low affinity for their counter receptors [37], a fact that may be exploited by viruses which can evolve a higher affinity for these receptors in order to invade the host cell [38]. We have previously identified the specific codon sites that have undergone adaptive changes in CD2 in the bovine lineage and revealed that they are almost exclusively limited to the extracellular portion of the molecular structure [4]. We speculated that a pathogen or more specifically, a virus interaction could have driven the rapid evolution observed in a region of the extracellular domain not involved in CD58/48 binding [4].

**Figure 1:**
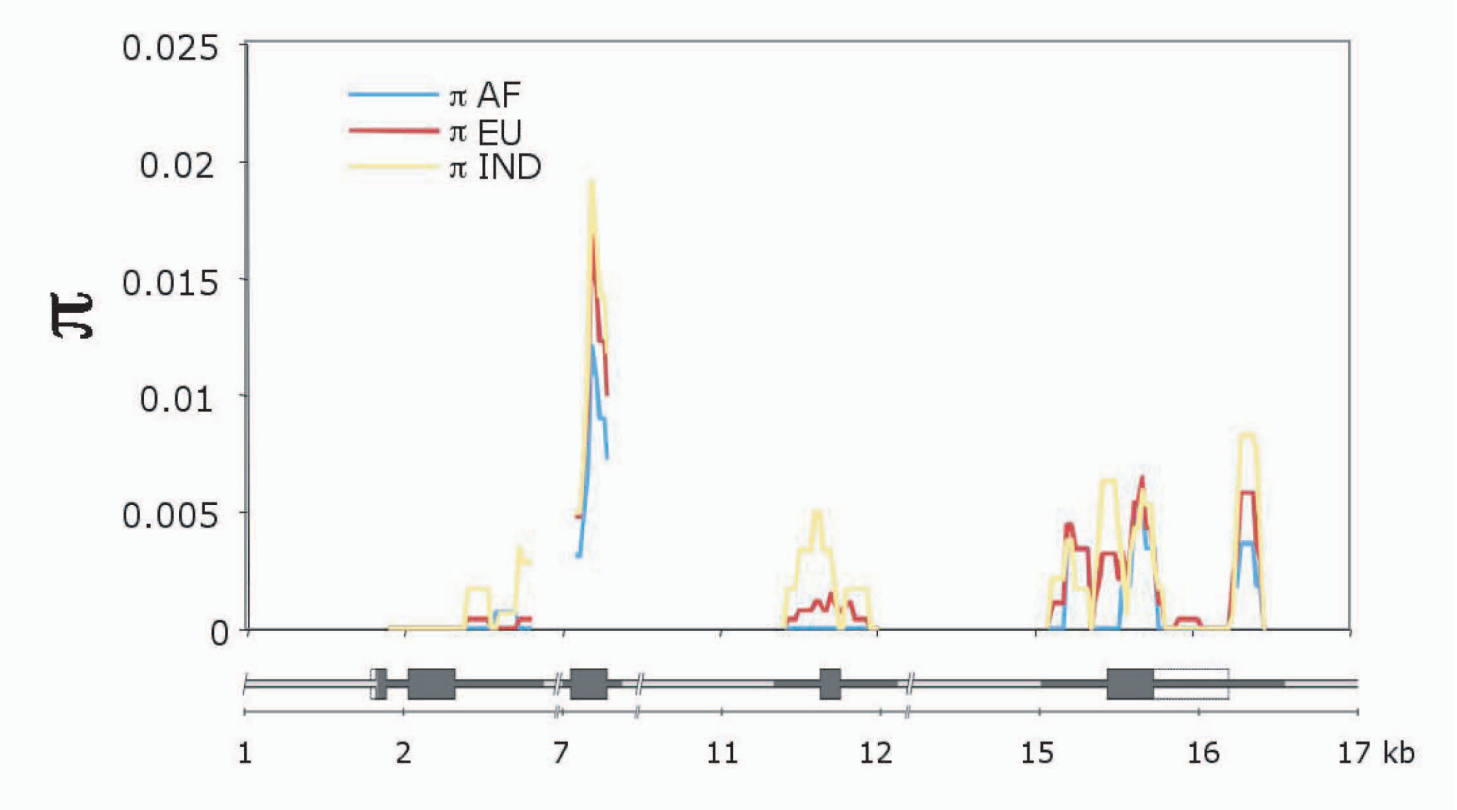
**Sliding window analysis of per-site sequence diversity (π) within each population for resequenced regions of CD2.** A window size of 150 bp and a slide of 25 bp were used. Regions sequenced for each locus are denoted as filled and exons as thicker rectangles in the scaled locus representation underneath. Plots are colour coded with reference to population (blue, African *Bos taurus*; red, European *Bos taurus*; yellow, *Bos indicus*). Note the peak in diversity within each population at exon 3.

Here we identified a significant Fay and Wu's *H *value within the European sample, indicating an excess of derived variants (P < 0.05; Table 1). This significance was robust to imposition of a domestication/post domestic expansion demographic model on test simulations; and using these as a null the African sample attained significance also. More strikingly, bovine CD2 displayed high coding polymorphism. Eleven nonsynonymous SNPs were present within the sampled populations, and there were an additional three nonsynonymous substitutions compared to the outgroup species, plains bison. Seven of the eleven within-cattle nonsynonymous SNPs and all of the outgroup nonsynonymous substitutions are located in the extracellular domain of CD2, a region which we have previously shown to be under adaptive evolution in cattle [4]. This genomic region (exon 3) displayed a clear peak in sequence diversity (Figure 1). This is reinforced by a visual inspection of haplotypes; six of these nonsynonymous changes lie within 130 bp and segregate in two strikingly divergent exon 3 haplotypes which are common in all three populations.

**Figure 2:**
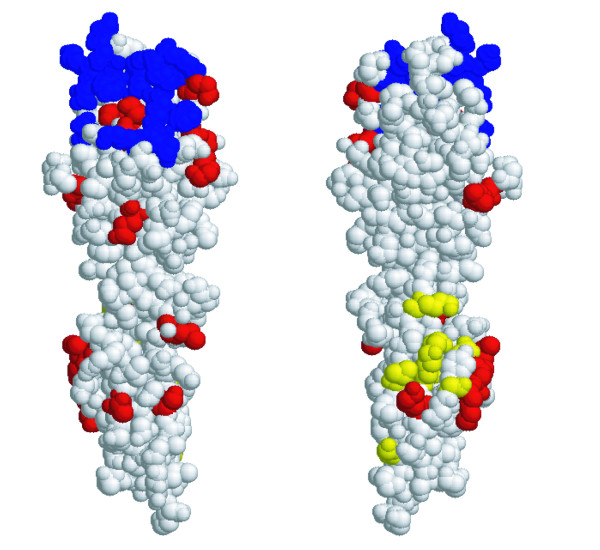
**Two alternative perspectives of the 3D structure of human CD2 extracellular domain.** Sites involved in CD58/CD48 binding are shown in blue. Codon sites (amino acids) predicted to be subject to positive selection from inter-specific comparison are shown in red. Yellow sites indicate the orthologous positions of nonsynonymous SNPs occurring within cattle populations. The structure was displayed using RasMol v2.7.2.1.1.

The locations of the seven extracellular domain nonsynonymous SNPs occurring within cattle populations were plotted on the structure of human CD2 based on their orthologous positions. It was noted that these sites appeared to cluster together in a particular region of the extracellular domain in the C2-set immunoglobulin domain (Figure 2). The median Cα distance between residues where nonsynonymous changes are observed within bovine populations was calculated as 11.12 Å. In comparison, the median distance between all residues in the structure human CD2 extracellular region was 24.47 Å. In simulations, there were only 16/1000 cases where the median distance of seven randomly selected residues was lower than that of the median distance between the cattle nonsynonymous changes, indicating that these residues are non-randomly clustered on the structure of CD2 (P = 0.016).

PolyPhen [19] predicts two substitutions, K180M and S189K, as "probably" and "possibly" damaging, respectively. The SIFT program [18] also predicts K180M, plus T156M, and V197M as likely to affect protein function. These variants each form part of the cluster haplotype of coding changes in exon 3 that are situated together on the surface of the extracellular domain of the protein.

## Discussion

The close proximity and subsequent migrations of humans and animals, following domestication, caused fundamental changes in the epidemiology of many infectious diseases [2]. Different cattle populations have been exposed differently to a variety of pathogens, such as rinderpest in early Asian populations [39] or trypanosomasis in sub-Saharan African populations [40]. Screening the genomes of domesticated animals for signatures of selection, particularly in relevant immune genes, may help pinpoint those genes that have been most important in the evolution of differential disease susceptibilities.

We queried whether six genes which have signatures suggestive of positive selection between species may have experienced continued selective pressure at the population level [6,7,41]. Notably, these genes were also chosen on the basis of having been previously implicated in susceptibility to bacterial, parasitic or viral infections. We re-sequenced each in 39 individuals comprising three distinct continental cattle populations. Several test statistics within the compilation of locus-population combinations yield evidence that recent adaptive change may have imprinted patterns of diversity around these genes. There is varying (but incomplete) concordance across tests. This is unsurprising as the statistics examine different aspects of locus diversity and are sensitive to selective processes that vary, for example in intensity or time depth [42]. The results fall into two interesting categories: first, outlying test statistics of allele frequency spectra in cytokines that imply the imprint of adaptive change but which are not associated with appreciable protein sequence polymorphism; and secondly, receptors which display coding variation suggestive of allelic functional variation, but which give less significant summary statistic values.

Of the latter loci, TYROBP gives the strongest statistical departure from neutrality, and has a derived nonsynonymous mutation almost fixed within European and African *B. taurus *cattle with predicted impact. In humans, ART4 encodes the Dombrock blood group glycoprotein [31]. It has been speculated that the polymorphic nature of this molecule in humans may be driven by an evolutionary advantage due to mutation of the RGD motif that may influence adhesion of the malaria parasite to erythrocytes [31]. Cattle do not appear to be polymorphic at the position responsible for the Do^a^/Do^b ^antigenicity in humans, but are polymorphic at the position associated with the Jo^a ^antigen, suggesting a structural or functional impact [32]. However, of these three, the most interesting evidence arises with CD2.

CD2 is a natural killer-cell and T-cell surface molecule that stabilizes interactions with antigen-presenting cells [34]. CD2 diversity gives outlying scores for Fay and Wu's *H *in both European and African *B. taurus *under a domestication-modeled simulation and within Europe under a standard coalescent model. Taken alone, these scores are not secure indications of recent adaptive evolution at this locus, but a closer look at the pattern of derived polymorphism underlying them gives several layers of additional support.

Firstly, CD2 was found to have a high number (eleven) of nonsynonymous changes within cattle (three between cattle and the outgroup species, plains bison). The majority of these are localized in the extracellular domain of the molecule, a region with a strong signal of positive selection (from inter-specific comparison), which may be driven by a host-pathogen genetic conflict [4]. Secondly, six of these changes are derived, located within a narrow window within exon 3 and define a divergent haplotype that appears at appreciable frequency in each of the three sampled populations. This region yields a signal of selection in sliding window analysis and has a peak of sequence diversity. Thirdly this genetically divergent haplotype is likely also to be functionally divergent. Three of these clustered substitutions are predicted to have phenotypic impact from biochemical and comparative information. Lastly, examination of a predicted 3D structure of the extracellular domain (Figure 2) showed that the seven substitutions clustered spatially – a configuration confirmed to be unlikely to be random by bootstrapping.

Taken together, these observations provide population genetic inference of adaptive evolution at the CD2 locus, possibly driven by receptor-pathogen interactions. Note that several similar receptors to CD2 are known to be exploited by viruses in order to invade the host cell; for example, the CD2 family member, SLAM, is bound by the measles virus [38].

Within the three cytokines sequenced, the strongest population genetic signatures of selection occur at the closely linked IL5 and IL13 loci within the African *B. taurus *population sample. Fay and Wu's *H *(IL5) plus Tajima's *D*, Fu and Li's *D *and *F *(IL13) values all show highly significant departures from the neutral model. Interestingly, these two loci comprise part of a cytokine cluster on chromosome 7 (syntenic with human chromosome region 5q31–33) which has key involvement in the Th2 mediated immune response and consequently, susceptibility to extracellular parasitic infections. There are well described and considerable parasitic challenges particular to African livestock; for example, non-indigenous breeds continue to be excluded from parts of West Africa by endemic trypanosomiasis [10]. Both of these loci show some reduction in nucleotide diversity within Africa (as does IL2) relative to the other populations but a focus of positive selection within each region is not easily localized from the data. Also, each interleukin sequenced showed a dearth of nonsynonymous change. IL5 and IL13 (along with IL4) are known to be influenced by *cis *factors over a 120 Kbp region and the focus of selection may be a remote regulatory sequence.

## Conclusion

This study alludes to two generalities. First, we show some correspondence between inference of selection from intra-species variation and that from the million year-order comparison between species. This hints that the diversity that was a matter of life and death for bovine antecedents has also been important in more recent eras, and probably continues to matter today. Second, we see contrasting patterns of diversity between the three signaling molecules (IL2, IL5 and IL13), which have outlying allele frequency spectra but insubstantial nonsynonymous polymorphism and three cell-surface receptors (TYROBP, ART4 and CD2), which interact directly with the extracellular environment and were observed to have significant coding polymorphism, giving some suggestion of adaptive history. This difference accords with prior work that records an increase of protein coding sequence adaptive events at the cellular periphery in humans, with more constraint on change at those loci which are internal and more central in interacting networks [43].

## Methods

### Re-sequencing strategy

We have re-sequenced almost all coding sequence, plus extensive intronic and intergenic regions from the IL13 (NM_174089), IL2 (NM_180997), IL5 (NM_173922), TYROBP (NM_174627), ART4 (XM_583864), and CD2 (NM_001011676) genes, in a panel of 18 European *Bos taurus*, 10 African *B. taurus *and 11 *B. indicus *cattle. These three population samples were drawn from 16 breeds of cattle (see Additional file 2). Breeds with known admixture were not included, as admixture of the scale in these breeds is a confounding factor in tests of selection. The orthologous regions were also re-sequenced in the outgroup species, plains bison (*Bison bison*). Primer details and PCR conditions are available for each gene sequenced (see Additional file 3). Samples were sequenced on the forward and reverse strands by Agowa, Germany . The regions re-sequenced for each gene are indicated figuratively (see Additional file 4). Sequence data from this article have been deposited with the EMBL/GenBank Data Libraries under accession no's. [GenBank:EF118301–EF118739; EU914955–EU914988; EU914991–EU915048].

### SNP detection and sequence analysis

Sequence assembly and SNP analysis was carried out using the Phred/Phrap/Consed/Polyphred pipeline . Bases were called and sequences assembled into contigs using Phred v0.020425.c [44,45] and Phrap v0.990319. Default parameters were used except for the forcelevel flag, which was set to a maximum value of 10, reducing the stringency of matching required for final contig assembly, and allowing outgroup sequences to be incorporated in subsequent SNP detection. Polyphred v.5.0 was used for polymorphism detection [46,47]. Reads were compared to the UCSC genomic reference sequence, and the source flag was used to optimize heterozygote identification. SNPs were checked manually and verified by examining trace files in Consed [48]. Alignments of polymorphic positions for each gene are available (see Additional file 1). PHASE v.2.1 was used to reconstruct haplotypes [49]. Nucleotide positions with lower confidence in sequence quality, i.e. fewer than 20 individuals with high quality sequence were masked and excluded from further analysis.

### Neutrality tests

We carried out several tests of neutrality based on allele frequency spectra derived from the re-sequencing data. These statistics were calculated over the entire re-sequenced regions (Table 1) using DnaSP [50,51]. The Tajima's *D *test [15] compared the difference between the number of segregating sites, *S*, and the average number of pairwise differences, π. Negative values of Tajima's *D *are due to an excess of low frequency variants, which may be due to positive selection or population expansion.

Fu and Li's *D *and *F *statistics incorporate information from a genealogy constructed with the outgroup sequence, plains bison. External branches of this genealogy tend to be populated by more recent mutations. Fu and Li's *D *is based on the differences between *Se*, the total number of mutations in external branches of the genealogy, and *S*, the total number of mutations, while, their *F *test statistic is based on the differences between *Se*, the total number of mutations in external branches of the genealogy, and *π *[16].

The *H *statistic, developed by Fay and Wu, requires an outgroup sequence, and is based on the difference between two estimators of *θ *: π and *θ*_*H*_, which is based on the frequency of the derived variants [23]. Fu's *Fs *[17] is based on the probability of observing a greater number of alleles in the population than expected under neutrality. Either genetic hitch-hiking or recent population expansion can cause a significant result in this test. For all of these tests, negative values result from an excess of low frequency and/or derived polymorphisms, while a deficiency of low frequency alleles causes a shift towards positive values.

One problem with neutrality test statistics can be their sensitivity to different demographies. Consequently, to test the robustness of our findings, we examined an alternative demographic history for whole gene coalescent simulations. These were used to construct empirical null distributions of two complementary test statistics (Tajima's *D *and Fay and Wu's *H) *presuming a constant population size of 10^4 ^until 10,000 years ago, followed by an instant reduction to 10^3 ^and an exponential increase to a present day size of 10^5^, a scenario consistent with the domestication and subsequent expansion of livestock. Simulations were carried out using MS [52].

### Predictive polymorphism phenotyping

Predictive polymorphism phenotyping of nonsynonymous SNPs was carried out using the PolyPhen  and Sorting Intolerant From Tolerant (SIFT)  programs [18,19]. PolyPhen assesses a number of parameters including, amino acid physiochemical properties, the level of sequence conservation in homologous proteins, and the proximity to structural or functional domains, to predict functionally significant nonsynonymous SNPs. Similarly, the SIFT program uses an alignment of orthologous or paralogous sequences to assess the usual level of sequence conservation at a polymorphic site and thus the potential functional importance of a substitution.

### Non-random Clustering of CD2 nonsynonymous SNPs

The distance between the alpha carbon atoms of each residue (Cα distance) in the 3 dimensional structure of the human CD2 extracellular domain (1 HNF.pdb) was calculated using the BioShell program .

## Abbreviations

Å: Angstrom; ART4: ADP-ribosyltransferase 4; bp: base pair; Cα distance: the distance between the alpha carbon atoms of each residue; CD2: cluster of differentiation 2 (CD2); IL: interleukin; kb: kilobase-pairs; KIR: killer-cell inhibitory receptor; NK: natural killer; π: nucleotide diversity; ω: ratio of nonsynonymous substitutions per nonsynonymous site to synonymous substitutions per synonymous site; S: segregating sites; Se: the total number of mutations in external branches of the genealogy; SIFT: Sorting Intolerant From Tolerant; SLAM: signaling lymphocytic activation molecule ; TYROBP: tyrosine kinase binding protein; UCSC: University of California Santa Cruz;

## Authors' contributions

ARF and DJL contributed equally to this work, both carried out data analysis and co-wrote the manuscript. ARF re-sequenced the genes studied in this project. CM assisted in SNP calling and in data analysis. DGB conceived of and oversaw the study and assisted in writing the manuscript.

## Supplementary Material

Additional File 1 **Alignments of polymorphic positions in re-sequenced bovine IL13, IL2, IL5, TYROBP, ART4 and CD2 genes.** Click here for file

Additional File 2 **Breed and country of origin information for each bovine sample.** Click here for file

Additional File 3 **Primer information for re-sequenced bovine genes.** Click here for file

Additional File 4 **Genomic structures of the genes encoding bovine A) Interleukin 13 (IL13); B) IL2; C) IL5; D) TYROBP; E) ART4 and F) CD2.** Exons are represented by shaded grey rectangles. Untranslated regions (UTRs) are similarly shown but are represented by a smaller height. Ns indicate regions not re-sequenced. Nonsynonymous SNPs ▲ (red), synonymous SNPs ■, and non-coding (intronic, upstream and downstream) SNPs | are shown. Click here for file

## References

[B1] Zeder MA, Emshwiller E, Smith BD, Bradley DG (2006). Documenting domestication: the intersection of genetics and archaeology. Trends Genet.

[B2] Pearce-Duvet JM (2006). The origin of human pathogens: evaluating the role of agriculture and domestic animals in the evolution of human disease. Biol Rev Camb Philos Soc.

[B3] White SN, Taylor KH, Abbey CA, Gill CA, Womack JE (2003). Haplotype variation in bovine Toll-like receptor 4 and computational prediction of a positively selected ligand-binding domain. Proceedings of the National Academy of Sciences of the United States of America.

[B4] Lynn DJ, Freeman AR, Murray C, Bradley DG (2005). A genomics approach to the detection of positive selection in cattle: adaptive evolution of the T-cell and natural killer cell-surface protein CD2. Genetics.

[B5] Hughes AL, Packer B, Welch R, Chanock SJ, Yeager M (2005). High level of functional polymorphism indicates a unique role of natural selection at human immune system loci. Immunogenetics.

[B6] Akey JM, Eberle MA, Rieder MJ, Carlson CS, Shriver MD, Nickerson DA, Kruglyak L (2004). Population history and natural selection shape patterns of genetic variation in 132 genes. PLoS Biol.

[B7] Nielsen R, Bustamante C, Clark AG, Glanowski S, Sackton TB, Hubisz MJ, Fledel-Alon A, Tanenbaum DM, Civello D, White TJ (2005). A scan for positively selected genes in the genomes of humans and chimpanzees. PLoS Biol.

[B8] Bradley DG, MacHugh DE, Cunningham P, Loftus RT (1996). Mitochondrial diversity and the origins of African and European cattle. Proc Natl Acad Sci USA.

[B9] Troy CS, MacHugh DE, Bailey JF, Magee DA, Loftus RT, Cunningham P, Chamberlain AT, Sykes BC, Bradley DG (2001). Genetic evidence for Near-Eastern origins of European cattle. Nature.

[B10] Freeman AR, Hoggart CJ, Hanotte O, Bradley DG (2006). Assessing the relative ages of admixture in the bovine hybrid zones of Africa and the Near East using X chromosome haplotype mosaicism. Genetics.

[B11] McKenzie GJ, Bancroft A, Grencis RK, McKenzie AN (1998). A distinct role for interleukin-13 in Th2-cell-mediated immune responses. Curr Biol.

[B12] Gibbs BF (2005). Human basophils as effectors and immunomodulators of allergic inflammation and innate immunity. Clin Exp Med.

[B13] Bancroft AJ, McKenzie AN, Grencis RK (1998). A critical role for IL-13 in resistance to intestinal nematode infection. J Immunol.

[B14] Fallon PG, Richardson EJ, McKenzie GJ, McKenzie AN (2000). Schistosome infection of transgenic mice defines distinct and contrasting pathogenic roles for IL-4 and IL-13: IL-13 is a profibrotic agent. J Immunol.

[B15] Tajima F (1989). Statistical method for testing the neutral mutation hypothesis by DNA polymorphism. Genetics.

[B16] Fu YX, Li WH (1993). Statistical tests of neutrality of mutations. Genetics.

[B17] Fu YX (1997). Statistical tests of neutrality of mutations against population growth, hitchhiking and background selection. Genetics.

[B18] Ng PC, Henikoff S (2001). Predicting deleterious amino acid substitutions. Genome Res.

[B19] Ramensky V, Bork P, Sunyaev S (2002). Human non-synonymous SNPs: server and survey. Nucleic Acids Res.

[B20] Loots GG, Locksley RM, Blankespoor CM, Wang ZE, Miller W, Rubin EM, Frazer KA (2000). Identification of a coordinate regulator of interleukins 4, 13, and 5 by cross-species sequence comparisons. Science.

[B21] Ellis MK, Zhao ZZ, Chen HG, Montgomery GW, Li YS, McManus DP (2007). Analysis of the 5q31 33 locus shows an association between single nucleotide polymorphism variants in the IL-5 gene and symptomatic infection with the human blood fluke, Schistosoma japonicum. J Immunol.

[B22] Gaffen SL, Liu KD (2004). Overview of interleukin-2 function, production and clinical applications. Cytokine.

[B23] Fay JC, Wu CI (2000). Hitchhiking under positive Darwinian selection. Genetics.

[B24] Diefenbach A, Tomasello E, Lucas M, Jamieson AM, Hsia JK, Vivier E, Raulet DH (2002). Selective associations with signaling proteins determine stimulatory versus costimulatory activity of NKG2D. Nat Immunol.

[B25] Lanier LL, Corliss BC, Wu J, Leong C, Phillips JH (1998). Immunoreceptor DAP12 bearing a tyrosine-based activation motif is involved in activating NK cells. Nature.

[B26] Sjolin H, Tomasello E, Mousavi-Jazi M, Bartolazzi A, Karre K, Vivier E, Cerboni C (2002). Pivotal role of KARAP/DAP12 adaptor molecule in the natural killer cell-mediated resistance to murine cytomegalovirus infection. J Exp Med.

[B27] Mohamadzadeh M, Coberley SS, Olinger GG, Kalina WV, Ruthel G, Fuller CL, Swenson DL, Pratt WD, Kuhns DB, Schmaljohn AL (2006). Activation of triggering receptor expressed on myeloid cells-1 on human neutrophils by marburg and ebola viruses. J Virol.

[B28] Hao L, Nei M (2004). Genomic organization and evolutionary analysis of Ly49 genes encoding the rodent natural killer cell receptors: rapid evolution by repeated gene duplication. Immunogenetics.

[B29] Sambrook JG, Bashirova A, Palmer S, Sims S, Trowsdale J, Abi-Rached L, Parham P, Carrington M, Beck S (2005). Single haplotype analysis demonstrates rapid evolution of the killer immunoglobulin-like receptor (KIR) loci in primates. Genome Res.

[B30] Storset AK, Slettedal IO, Williams JL, Law A, Dissen E (2003). Natural killer cell receptors in cattle: a bovine killer cell immunoglobulin-like receptor multigene family contains members with divergent signaling motifs. Eur J Immunol.

[B31] Gubin AN, Njoroge JM, Wojda U, Pack SD, Rios M, Reid ME, Miller JL (2000). Identification of the dombrock blood group glycoprotein as a polymorphic member of the ADP-ribosyltransferase gene family. Blood.

[B32] Reid ME (2003). The Dombrock blood group system: a review. Transfusion.

[B33] Brossay A, Hube F, Moreau T, Bardos P, Watier H (2003). Porcine CD58: cDNA cloning and molecular dissection of the porcine CD58-human CD2 interface. Biochem Biophys Res Commun.

[B34] Selvaraj P, Plunkett ML, Dustin M, Sanders ME, Shaw S, Springer TA (1987). The T lymphocyte glycoprotein CD2 binds the cell surface ligand LFA-3. Nature.

[B35] Shimojima M, Nishimura Y, Miyazawa T, Kato K, Nakamura K, Izumiya Y, Akashi H, Tohya Y (2002). A feline CD2 homologue interacts with human red blood cells. Immunology.

[B36] Kato K, Koyanagi M, Okada H, Takanashi T, Wong YW, Williams AF, Okumura K, Yagita H (1992). CD48 is a counter-receptor for mouse CD2 and is involved in T cell activation. J Exp Med.

[B37] Dustin ML, Golan DE, Zhu DM, Miller JM, Meier W, Davies EA, Merwe PA van der (1997). Low affinity interaction of human or rat T cell adhesion molecule CD2 with its ligand aligns adhering membranes to achieve high physiological affinity. J Biol Chem.

[B38] Wang J (2002). Protein recognition by cell surface receptors: physiological receptors versus virus interactions. Trends Biochem Sci.

[B39] Spinage CA (2003). Cattle plague: a history.

[B40] Gifford-Gonzalez D (2000). Animal Disease Challenges to the Emergence of Pastoralism in Sub-Saharan Africa. African Archaeological Review.

[B41] Gilbert SL, Dobyns WB, Lahn BT (2005). Genetic links between brain development and brain evolution. Nat Rev Genet.

[B42] Nielsen R, Hellmann I, Hubisz M, Bustamante C, Clark AG (2007). Recent and ongoing selection in the human genome. Nat Rev Genet.

[B43] Kim PM, Korbel JO, Gerstein MB (2007). Positive selection at the protein network periphery: evaluation in terms of structural constraints and cellular context. Proc Natl Acad Sci USA.

[B44] Ewing B, Green P (1998). Base-calling of automated sequencer traces using phred. II. Error probabilities. Genome Res.

[B45] Ewing B, Hillier L, Wendl MC, Green P (1998). Base-calling of automated sequencer traces using phred. I. Accuracy assessment. Genome Res.

[B46] Stephens M, Sloan JS, Robertson PD, Scheet P, Nickerson DA (2006). Automating sequence-based detection and genotyping of SNPs from diploid samples. Nat Genet.

[B47] Nickerson DA, Tobe VO, Taylor SL (1997). PolyPhred: automating the detection and genotyping of single nucleotide substitutions using fluorescence-based resequencing. Nucleic Acids Res.

[B48] Gordon D, Abajian C, Green P (1998). Consed: a graphical tool for sequence finishing. Genome Res.

[B49] Stephens M, Smith NJ, Donnelly P (2001). A new statistical method for haplotype reconstruction from population data. Am J Hum Genet.

[B50] Rozas J, Rozas R (1999). DnaSP version 3: an integrated program for molecular population genetics and molecular evolution analysis. Bioinformatics.

[B51] Rozas J, Sanchez-DelBarrio JC, Messeguer X, Rozas R (2003). DnaSP, DNA polymorphism analyses by the coalescent and other methods. Bioinformatics.

[B52] Hudson RR (2002). Generating samples under a Wright-Fisher neutral model of genetic variation. Bioinformatics.

