# Supplementary figures and images for: Detecting the effects of selection at the population level in six bovine immune genes
Source: BMC Genet. 2008 Oct 6;9:62. doi: 10.1186/1471-2156-9-62 (PMC2576349; doi:10.1186/1471-2156-9-62)

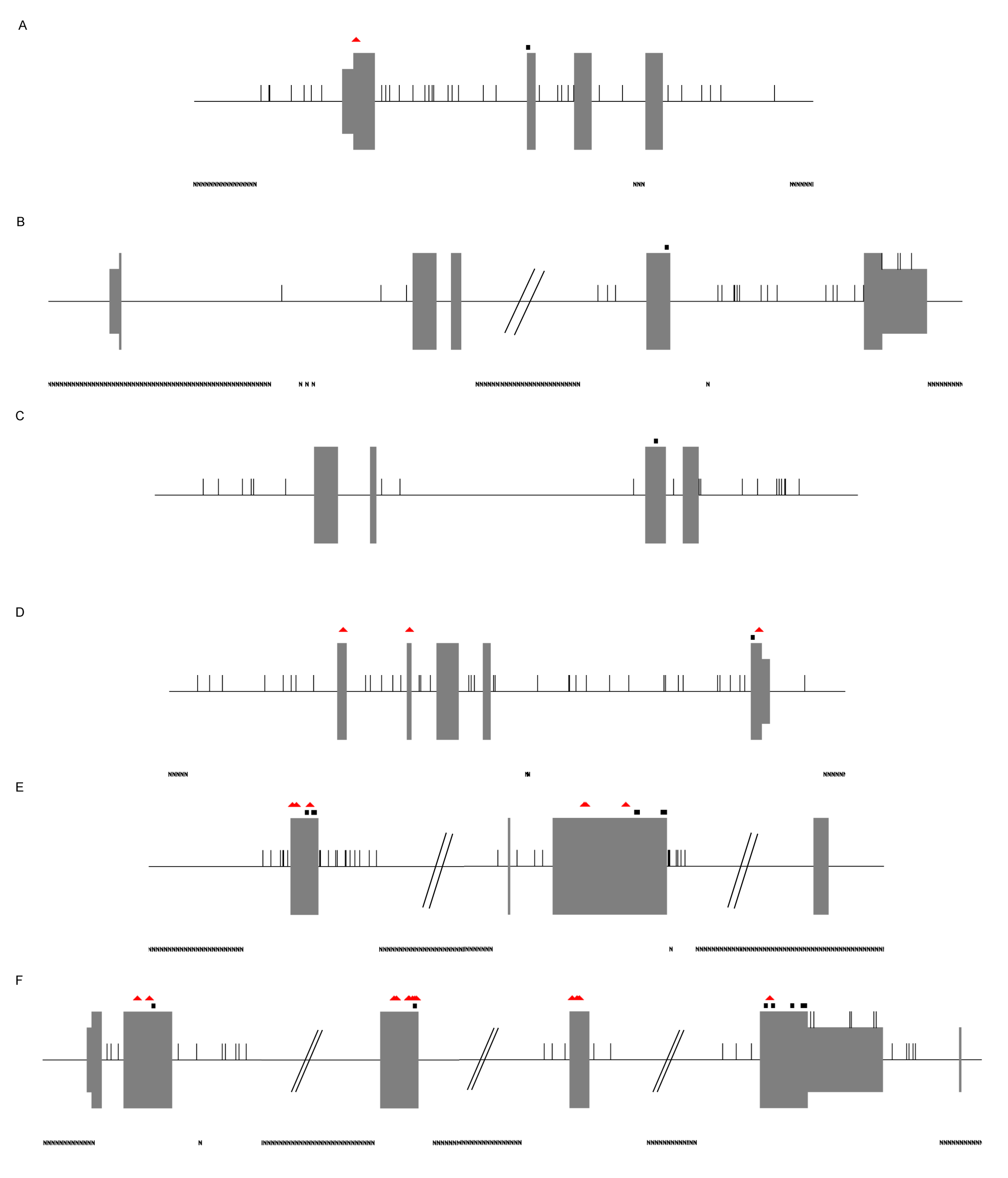

Supplement: Additional File 4 — Genomic structures of the genes encoding bovine A) Interleukin 13 (IL13); B) IL2; C) IL5; D) TYROBP; E) ART4 and F) CD2. Exons are represented by shaded grey rectangles. Untranslated regions (UTRs) are similarly shown but are represented by a smaller height. Ns indicate regions not re-sequenced. Nonsynonymous SNPs ▲ (red), synonymous SNPs ■, and non-coding (intronic, upstream and downstream) SNPs | are shown. [file 1471-2156-9-62-S4.tiff]
